# SWAT 119: the effectiveness of a Thank You card to improve trial follow-up; a randomised study within a trial (SWAT)

**DOI:** 10.1186/s13063-026-09636-0

**Published:** 2026-03-31

**Authors:** S. Zahra, F. Wiggins, T. Yakimova, K. Baird, I. Chetter, C. Fairhurst, A. Mott, S. Swan, J. Wilkinson, C. E. Arundel

**Affiliations:** 1 York Trials Unit, Department of Health Sciences, Faculty of Science, University of York, York, UK; 2 University of Hull, Hull, HU6 7RX UK; 3 Hull York Medical School, Hull, HU6 7RX UK; 4 Hull University Teaching Hospitals NHS Trust, Hull, HU3 2JZ UK

**Keywords:** SWAT, Study within a trial, Retention methods, Embedded randomised controlled trial, Thanks

## Abstract

**Background:**

Retention in randomised controlled trials is crucial to maximising study power and generalisability. A Study Within A Trial (SWAT) evaluated if sending a Thank You card improved 6-month questionnaire return rates in the SWHSI-2 trial.

**Methods:**

A two-arm SWAT, using 1:1 (intervention:control) allocation, embedded within the SWHSI-2 trial. The primary outcome was the difference in the return rate of the 6-month questionnaire. Secondary outcomes were the difference in return rate of the 12-month questionnaire, questionnaire completeness, need for a reminder, and cost. The primary analysis was conducted using mixed-effects logistic regression adjusted for main trial allocation as a fixed effect and site as a random effect. Random-effects meta-analysis combined all available data for this intervention.

**Results:**

A total of 560 participants were included in the SWAT. There was no difference in the 6-month questionnaire return rate between the Thank You card group and the no Thank You card group (OR 1.04, 95% CI 0.73 to 1.50, *p* = 0.87). The 12-month retention rate was slightly higher in the Thank You card group, but the difference was not statistically significant (OR 1.15, 95% CI 0.81 to 1.62, *p* = 0.43). Findings were very similar in sensitivity analyses accounting for intervention participants who did not receive their card. There was no evidence of difference for any of the remaining secondary outcomes. Meta-analysis of the 12-month return rate suggests that Thank You cards may provide slight improvements in questionnaire response rates; however there is uncertainty in this estimate (OR 1.07, 95% CI 0.79 to 1.45).

**Conclusion:**

It remains unclear if a Thank You card increased the rate of 6-month follow-up questionnaire completion in the SWHSI-2 trial. This is further amplified by the limited number of SWAT replications completed to date and included in the meta-analysis (*n*=2). The SWATs to date have primarily been undertaken in trials with a predominantly older, white, male, population. Further SWAT replications are therefore required in other populations to ensure generalisability of a cumulative SWAT finding.

**Trial registration:**

*Host Trial:* ISRCTN26277546. Prospectively registered on 25^th^ March 2019.

*SWAT:* MRC Hub for Trials Methodology Research SWAT repository #119 can be found at: https://www.qub.ac.uk/sites/TheNorthernIrelandNetworkforTrialsMethodologyResearch/FileStore/Filetoupload,959362,en.pdf.

## Background

Retaining participants to randomised controlled trials (RCTs) is crucial in determining study success [1]. Retention challenges revolve mainly around study duration, the intervention regime and personal circumstances of study participants [2].

Various retention strategies are used to maintain participants’ engagement throughout the trial. One way to test these retention strategies is through methodological Studies Within A Trial (SWATs) [3]. A SWAT is a self-contained research study embedded within a host trial with the aim of evaluating or exploring alternative ways of delivering or organising a particular trial process [4]. A recent systematic review of 70 retention SWATs encouraged evaluating embedded retention strategies within a host trial to develop robust evidence to maximise participant retention across trials [1].

Retention strategies such as newsletters or incentives routinely include an element of thanks within them either directly or implicitly. ‘Thanking’ participants as a retention strategy is based on the psychology of positive reinforcement whereby reinforcing a behaviour aims to promote replication at subsequent timepoints [5].

Sending a ‘Thank You’ card to study participants to thank them for their time, effort and study participation could potentially be a cheap and efficient way of successfully retaining participants in a study. A previous SWAT was conducted in which participants were sent a Thank You card 9 months after treatment delivery to evaluate their impact on the 1-year post treatment retention rate [6]. The SWAT found no evidence of a difference in retention rates between groups [6] but recommended further replications of the SWAT to produce high certainty evidence for this intervention.

This paper presents the second replication and initial meta-analysis of a ‘Thank You card’ SWAT embedded within a host trial to provide further evidence with regards to the following question: Does giving participants a Thank You card increase retention rate at the 6-month follow-up time point compared to not receiving a Thank You card?

## Methods

### Design

A two-arm, parallel group, randomised controlled SWAT was undertaken within the SWHSI-2 trial allocating participants 1:1 to the Thank You card intervention or no Thank You card control. The SWAT protocol (number 119) can be found at: https://www.qub.ac.uk/sites/TheNorthernIrelandNetworkforTrialsMethodologyResearch/FileStore/Filetoupload,959362,en.pdf.

The SWHSI-2 trial protocol has been previously published [7]. In brief, SWHSI-2 was a pragmatic, multicentre, randomised controlled trial to assess the clinical and cost-effectiveness of negative pressure wound therapy versus usual care for surgical wounds healing by secondary intention. The primary outcome was time to wound healing in days since randomisation and secondary outcomes were clinical events (e.g., return to hospital, amputation), wound infection, wound pain, quality of life, and resource use. Outcomes were collected through wound-related weekly assessment calls to participants, wound photographs (at baseline and healing) and via postal or telephone follow-up case report forms completed by/with participants [7].

The SWAT was considered low risk and was approved by the Research Ethics Committee Yorkshire and Humber – Leeds East (19/YH/0054).

Participants were not informed of this SWAT and so did not provide informed consent for their involvement. This is justified, supported by guidance [4] due to the low risk imposed by SWATs, the limited additional burden to participants, and also due to the fact that obtaining consent may both confuse participants and also impact on their behaviour thus confounding the SWAT evaluation.

### Participants

The SWAT was embedded in the SWHSI-2 trial formally on 24.01.2020 with the first Thank You card sent on 01.03.2020. All participants recruited into the SWHSI-2 trial, who remained as fully participating (i.e. had not fully withdrawn, withdrawn from postal follow-up or had died) and who were yet to reach the 4-month time point were eligible for the SWAT.

There were no additional inclusion or exclusion criteria.

### Intervention

The SWAT intervention was a Thank You card (see Appendix 1) sent to study participants at the start of the 4^th^ and 9^th^ month following randomisation in advance of receiving the 6 and 12-month follow-up questionnaires. The decision to administer the Thank You card at these time points was multifaceted: i) administration was at the midpoint between assessment timepoints thus corresponding to previous replications of this SWAT [6] and ii) This timing was anticipated to both facilitate the intended positive behaviour reinforcement of the intervention and also to act as a reminder to participants that they would receive a questionnaire in due course.

As per earlier replications of this SWAT [6], the card included a standardised message from the trial team and was not individually addressed or signed. Cards were mailed to participants in a white envelope with the address handwritten using second class Royal Mail Mailmark franking.

The control was ‘no Thank You card’.

### Outcomes

Primary outcome:

- Difference in retention rate at 6 months of participants who received the Thank You card vs those who did not receive a Thank You card. Retention rate was defined as the proportion of participants who completed and returned the questionnaire booklet at the 6 months outcome timepoint.

Secondary outcomes:

- Completeness of response, defined as the proportion of the questionnaire completed, at 6 and 12 months
- Need for a reminder letter, defined as the proportion of participants sent a reminder letter following non-response to the initial questionnaire, at 6 and 12 months
- Retention rate at 12 months
- Cost per additional participant retained (if effect was positive) calculated as the total SWAT cost divided by the number of additional participants retained.

No changes were made to the SWAT outcomes once the SWAT started.

### Sample size

The SWAT sample size was dependent on the number of participants actively participating within the host trial (SWHSI-2), hence a formal power calculation to determine sample size was not conducted, in accordance with accepted SWAT methodology [4]. Only those participants who were yet to reach the 4-month time point in the trial upon SWAT implementation, and were still in active participation, were included.

### Randomisation

Participants were allocated 1:1 using block randomisation, stratified by the host trial’s treatment arm, using randomly varying block sizes. The allocation sequence was generated using Stata v15 (StataCorp, 4905 Lakeway Drive, College Station, TX, 77845 USA) by the SWHSI-2 statistician based at York Trials Unit, University of York who was not involved with the follow-up of participants, or the printing and mailing of the Thank You cards.

### Blinding

Given the nature of the SWAT, it was not possible to blind the central coordinating team to participant allocation. Participants were not informed about their explicit participation in the SWAT and so were blinded to SWAT allocation.

### Statistical analysis

A CONSORT diagram depicts the flow of participants through the SWAT [8]. Participant baseline characteristics are summarised descriptively by group. All statistical tests were two-sided, with a 5% significance level. Parameter estimates are given with associated 95% confidence intervals (CI) and *p*-values. Participants were analysed in the groups to which they were randomised, following the principles of intention-to-treat.

Primary outcome analysis: mixed-effects logistic regression was used to assess the difference in the primary outcome (binary outcome, response rate at 6-month follow-up), adjusting for main trial allocation (fixed effect) and site (random effect). The absolute difference in return rates was estimated using a two-sample test of proportions.

Secondary outcome analysis: mixed-effect regression with main trial allocation as a fixed effect and site as a random effect was used to analyse secondary outcomes (linear regression for continuous outcome of completeness of questionnaire completion and logistic regression for the binary categorical outcome of the necessity for a reminder notice). The difference in costs per retained participant was calculated including direct (printing and postage) and indirect (staff time) costs.

A sensitivity analysis was conducted using CACE analysis to account for non-compliance with the SWAT intervention [9].

A random-effects meta-analysis compared the rates of questionnaire return at the 12-month follow-up, combining data from this SWAT and a similar one embedded in the DISC trial [6].

## Results

**Fig. 1:**
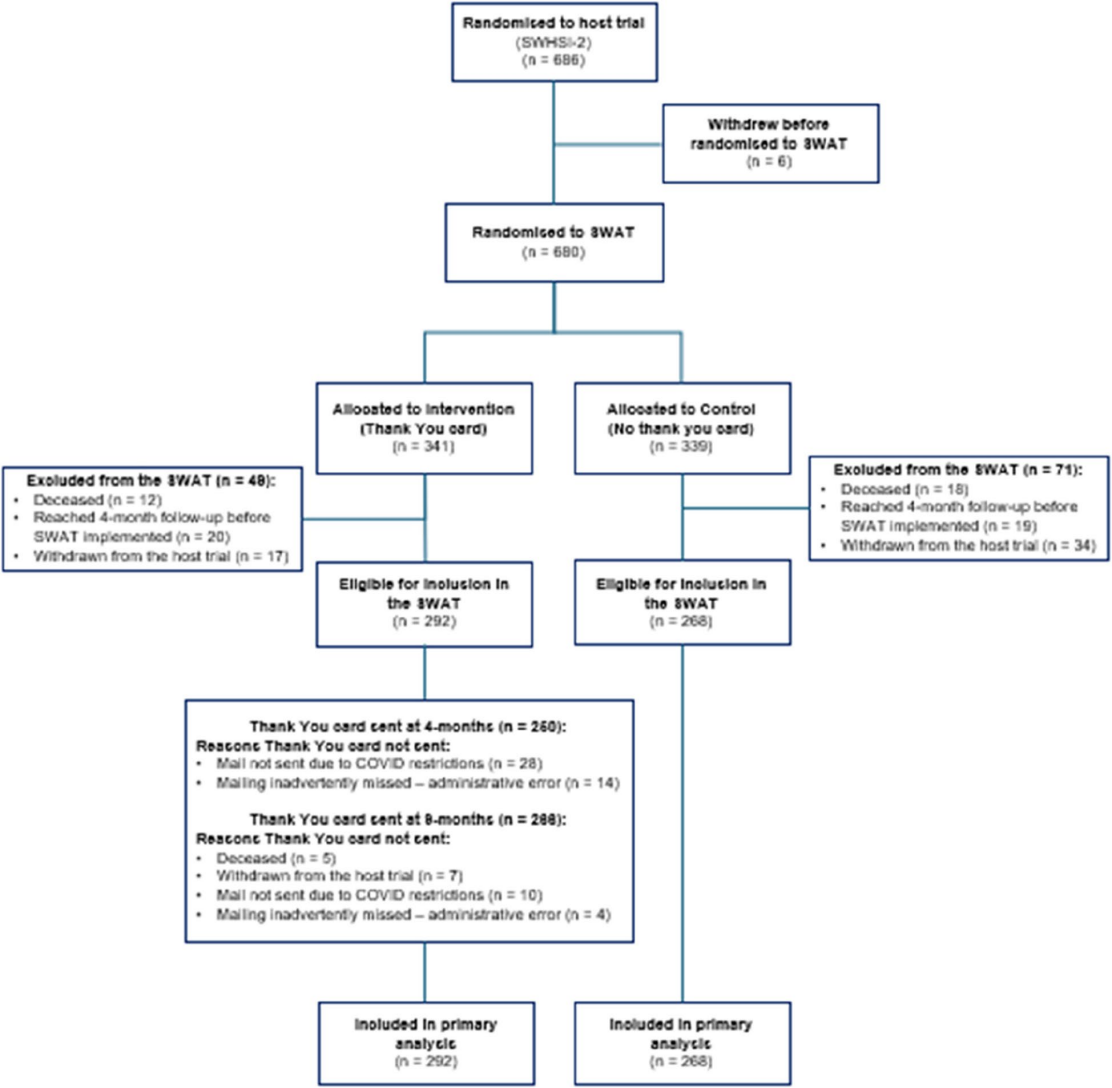
Study flow diagram displaying the flow of the participants through the SWAT

The Thank You card SWAT ran between 24.01.2020 (SWAT implementation date) and 13.01.2024 (final follow-up for the host trial). Overall, 686 participants were recruited in the main SWHSI-2 trial. For logistical reasons, randomisation to the SWAT occurred shortly after enrolment in the main trial so allocations were generated for 680 participants (six participants withdrew from the host trial immediately after randomisation): *n*=341 (50.1%) to the Thank You card Intervention group, and *n*=339 (49.9%) to the No Thank You card Control group. However, only 560 participants were eligible for inclusion in the SWAT at the point it was implemented (i.e. reached 4-month follow-up after SWAT implementation and had not died, fully withdrawn or withdrawn from postal follow-up): 292 were randomised to receive a Thank You card, and 268 not to receive a Thank You card. Figure 1 shows study flow diagram displaying the flow of the participants through the SWAT.

Table 1 describes the baseline characteristics of included participants. The average age of was 62 years, 75% were male and participants were predominantly white. The SWAT intervention groups were similar in terms of main trial allocation, age and ethnicity, but there was a slightly higher proportion of males in the Thank You card group than the no Thank You card group.

**Table 1:** Baseline characteristics of the SWAT participants

	**Intervention** **(Thank You card)** **n = 292**	**Control** **(No Thank You card)** **n = 268**	**Total** **n = 560**
**Main trial allocation, n (%)**			
NPWT	146 (50.0)	131 (48.9)	277 (49.5)
Usual care	146 (50.0)	137 (51.1)	283 (50.5)
**Age**			
N	291	268	559
Mean (SD)	62.4 (11.7)	61.8 (13.6)	62.1 (12.6)
Range	29.2 - 90.7	21.7 - 87.6	21.7 - 90.7
**Gender, n (%)**			
Male	231 (79.1)	189 (70.5)	420 (75.0)
Female	60 (20.5)	79 (29.5)	139 (24.8)
Missing	1 (0.3)	0 (0.0)	1 (0.2)
**Ethnicity, n (%)**			
White	272 (93.2)	242 (90.3)	514 (91.8)
Asian or Asian British	7 (2.4)	16 (6.0)	23 (4.1)
Black or Black British	8 (2.7)	7 (2.6)	15 (2.7)
Other	3 (1.0)	1 (0.4)	4 (0.7)
Missing	2 (0.7)	2 (0.7)	4 (0.7)

Not all intervention group participants received the Thank You card at month 4 for the following reasons: it was due during the first COVID-19 national lockdown in 2020 at a time when research staff members were unable to access their office building to send post to participants (*n*=28); or it was not sent in error (*n*=14) due to administrative error. Further at month 9, 26 intervention participants were not sent the card: died/withdrawn from main trial before this point (n=12), COVID-19 restrictions (*n*=10), and not sent in error due to administrative error (*n*=4).

There was no difference in the likelihood of returning the questionnaire at the 6- or 12-month follow-up between participants who received a Thank You card and those who did not (6-month: 67.8% vs 67.2%; OR 1.04, 95% CI 0.73 to 1.50, p = 0.82; 12-month: 61.3% vs 58.2%; OR 1.15, 95% CI from 0.81 to 1.62, *p* = 0.43; Table 2). The absolute difference in return rates was 0.6% (95% CI from −7.1% to 8.4%, *p* = 0.87) and 3.1% (95% CI from −5.0% to 11.2%, *p* = 0.46), respectively.

**Table 2:** Primary and secondary retention outcome at 6 and 12 months

	**Primary analysis**		
	**Intervention** **(Thank you card)** **N=292**	**Control** **(No Thank you card)** **N=268**	**p-value**
**6-month follow-up**			
Returned questionnaire, n (%)	198 (67.8)	180 (67.2)	
Adjusted odds ratio (95% CI)	1.04 (0.73 to 1.50)		0.82
Absolute difference in return rates (95% CI)	0.6% (−7.1% to 8.4%)		0.87
**12-month follow-up**			
Returned questionnaire, n (%)	179 (61.3)	156 (58.2)	
Adjusted odds ratio (95% CI)	1.15 (0.81 to 1.62)		0.43
Absolute difference in return rates (95% CI)	3.1 (−5.0% to 11.2%)		0.46

In sensitivity analyses, the CACE estimates of the difference in response at 6 and 12 months between the SWAT groups were similar to the main analyses (OR 1.07, 95% CI 0.70 to 1.63, *p*=0.76, and 1.17, 95% CI 0.80 to 1.71, *p*=0.42, respectively).

Amongst participants who returned the 6-month questionnaire, the average percentage of questionnaire completeness was slightly lower in the Thank You card group (mean 72.3, SD 35.0) than in the no Thank You card group (mean 77.0, SD 33.6). The adjusted mean difference (AMD) was −5.7 (95% CI −12.3 to 0.90, *p*=0.09). Similarly at 12 months, mean 58.1, SD 35.8 vs mean 60.2, SD 36.7, AMD −3.08, 95% CI −10.5 to 4.4, *p*=0.42.

At 6 months, 60.9% of participants required a reminder to be sent to complete the questionnaire (62.7% in the Thank You card group, and 59.0% in the no Thank You card group; OR 1.17, 95% CI 0.83 to 1.65, *p*=0.37). At 12 months, 63.0% of participants required a reminder to be sent to complete the questionnaire (61.3% in the Thank You card group, and 64.9% in the no Thank You card group; OR 0.85, 95% CI 0.60 to 1.21, *p*=0.37).

The total cost of sending the Thank You cards was £428.74 which equates to £0.75 per card (Table 3). As no statistically significant effect of the intervention was identified a cost per additional participant was not calculated.

**Table 3:** Costs associated with the Thank You card SWAT

Task	Total Cost	No cards involved	Cost per card
Printing cards^*^	£155.00	700	£0.22
Preparing cards^+^	£61.36	516	£0.12
Postage^!^	£212.38	516	£0.41
Total cost	£428.74	-	£0.75

^*^A total of 700 cards were ordered for the SWAT, sufficient to allow all SWHSI-2 Trial participants to be randomised into the study and to receive cards at both timepoints

+Preparation and packaging were completed by a University of York Grade 3 member of staff with the salary midpoint of the pay scale used for calculations. Preparation and packaging took approximately 259 minutes (4.33 hours) to complete at a rate of approximately 30 seconds per card

!Cards were sent to SWAT intervention participants via Royal Mail Mailmark franking at a cost of £0.41 per card

**Fig. 2:**
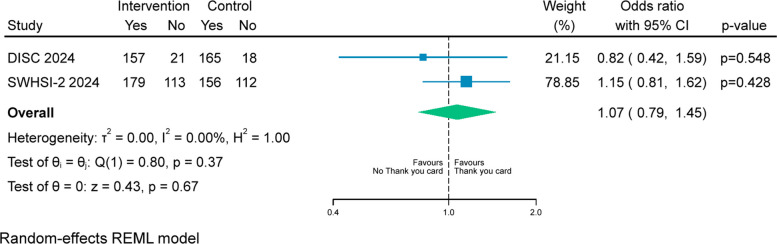
Retention rates at 12-month follow-up

A meta-analysis combining data from the SWHSI-2 and DISC trial SWATs was also performed (Fig. 2). Overall, there was no statistically significant difference in likelihood in return rates for patients who received a Thank You card at 12-month follow-up (OR 1.07, 95% CI 0.79 to 1.45, *p*=0.67).

### Harms

Data in relation to harms or unintended effects were not collected, nor were any noted during the conduct of this SWAT.

## Discussion

The current SWAT found no evidence of a difference in return rate of 6-month follow-up questionnaires between participants randomised to receive a Thank You card or not to receive a Thank You card. These findings are contrary to the findings of the DISC trial SWAT [6] which reported a higher proportion of participants in the control group (who did not receive a Thank You card) completed their DISC 1-year outcome assessment compared to those who were sent a card; however, like the SWHSI-2 trial SWAT, these findings were not statistically significant.

The proportion of participants requiring a reminder letter was similar in both groups at both timepoints, but levels of completeness of the questionnaire were slightly lower in the Thank You card group at both timepoints. The intervention was relatively low cost at £0.75 per card sent; the cost per additional participant retained was not calculated due to the absence of any evidence of effect.

Pooled data from SWHSI-2 and DISC trial SWAT found no difference between group retention rates at 12-months; however the effect estimate did slightly favour the intervention group. Given the limited evidence available around the specific effect of sending out a Thank You card for participant retention, further replication of this intervention is needed.

While this was a relatively low cost and easy to implement intervention, it is important to acknowledge any challenges to the delivery of this intervention for the benefit of those considering further replication. Trial teams should consider the staff time needed to prepare and send the thank you cards as this is an additional administrative task for the study. The impact of this was limited in this SWAT by using pre-printed cards, with additional time incurred largely in relation to the handwriting of envelopes. This could be minimised in future studies by utilising pre-printed participant address labels.

### Strengths and limitations

This SWAT has added to the existing evidence around Thank You card interventions initially established from a SWAT conducted in the DISC trial [6].

Several participants were unable to be sent their Thank You card due to COVID-19 restrictions limiting the trial teams’ access to their office to post them out at the time. In addition, the sending of 18 cards was inadvertently missed (14 at month 4, and 4 at month 9) due to administrative error. A CACE analysis was conducted to account for this non-compliance which resulted in a similar conclusion to the primary outcome.

The participants included within the SWAT for SWHSI-2 and DISC trials were representative of the overall trial population. The average age, proportion of male vs female participants and ethnicity proportions were not substantially different between the SWAT and host trials population. As the host trial and hence SWAT population largely comprised older Caucasian males, this may limit the generalisability and transferability of findings to different ethnic, gender and age groups, so caution must also be taken with the meta-analysis findings. Further replications in other populations are needed.

The intervention was delivered at month 4 and 9 following randomisation, i.e., 2 to 3 months before questionnaires were sent. There is potential that this timing may not have been optimal, and so as highlighted in the DISC SWAT [6], future replications should consider the optimum time point for intervention administration to either confirm or contrast with the existing evidence.

### Implications for trial practice and SWAT research

Given this is the second replication of a SWAT of this intervention, further replications are needed and so the intervention should currently only be used when being evaluated in a SWAT. Further evidence for intervention effectiveness is needed in populations other than white males and in a broader diversity of age groups to produce sufficient evidence to determine the role of this intervention on retention rates in RCTs more broadly.

## Conclusion

It remains unclear if Thank You cards increase the rate of follow-up completion. Further SWAT replications are therefore required, ideally in populations other than older, male, Caucasians, to provide additional evidence to distinguish conclusively whether distinct thanks within a RCT is an effective retention strategy.

## Data Availability

All data requests should be submitted to the corresponding author for consideration. Access to anonymised data may be granted following review.
